# Perineural Invasion in Breast Cancer: A Comprehensive Review

**DOI:** 10.3390/cancers17121900

**Published:** 2025-06-06

**Authors:** Hisham F. Bahmad, Carter Wegner, Joana Nuraj, Rima Avellan, Jeffrey Gonzalez, Teresita Mendez, Diana Jabbour, Carmen Gomez-Fernandez

**Affiliations:** 1 Department of Pathology and Laboratory Medicine, University of Miami Miller School of Medicine, Miami, FL 33136, USA; cgomez3@med.miami.edu; 2 Herbert Wertheim College of Medicine, Florida International University, Miami, FL 33199, USA; cwegn001@med.fiu.edu (C.W.); jnura002@med.fiu.edu (J.N.); ravel001@med.fiu.edu (R.A.); jgonz1074@med.fiu.edu (J.G.); tmend022@med.fiu.edu (T.M.); 3 Faculty of Medical Sciences, Lebanese University, Hadath Campus, Beirut 1003, Lebanon; diana.jabbour@outlook.com

**Keywords:** breast cancer, ductal carcinoma in situ, invasive ductal carcinoma, perineural invasion, perineural entrapment, tumor microenvironment, review

## Abstract

Perineural invasion (PNI) is a well-recognized histopathologic feature in multiple malignancies; however, its significance in breast cancer remains relatively underexplored. This review provides a synopsis of the current knowledge on PNI in breast cancer, discussing its histopathologic features, molecular mechanisms, diagnostic challenges, and clinical relevance. PNI is most frequently observed in high-grade invasive ductal carcinoma (IDC), particularly in triple-negative and HER2-positive subtypes. It is also seen in special histological subtypes such as mixed, metaplastic, and invasive micropapillary carcinomas. Mechanistically, PNI involves tumor–neural interactions, including neurotrophic factor signaling and epithelial–mesenchymal transition, contributing to tumor progression and potential locoregional recurrence (LRR). While PNI is linked to adverse prognosis in other tumors, its independent role remains unclear in breast cancer due to limited large-scale studies. Therefore, further investigation into its prognostic significance and potential therapeutic implications is needed. Future research should focus on refining diagnostic criteria and assessing targeted therapies to mitigate PNI-associated progression. This review summarizes the current knowledge on perineural invasion (PNI) in breast cancer, addressing its histological features, molecular mechanisms, diagnostic challenges, and clinical implications.

## Simple Summary

Perineural invasion (PNI) is a well-established histopathologic feature in cancer, but its significance in breast pathology remains underexplored. While PNI is understudied in breast cancer relative to other malignancies, some studies correlate it with higher tumor grade, increased locoregional recurrence risk, and potentially worse prognosis. This review underscores the need for standardized diagnostic criteria and further evidence to clarify PNI’s prognostic role and therapeutic potential in breast cancer.

## 1. Introduction

Among the various mechanisms of cancer propagation, perineural invasion (PNI) emerges as an important route for tumor metastasis, independent of lymphatic or vascular dissemination [1]. This phenomenon is not exclusive to cancer but can also be seen in benign pathological conditions [2]. Broadly defined as the presence of neoplastic cells within any nerve sheath layer (epineurium, perineurium, or endoneurium) or encircling at least 33% of the nerve circumference [3], the extent of nerve involvement in lesions exhibiting PNI varies.

Historically, PNI was described as tumor invasion in, around, or through nerves [4,5], but recent definitions adopt stricter criteria [1]. PNI is often observed as tumor–nerve contact within the perineurium without direct invasion, most frequently through complete or incomplete encirclement, and less commonly via sandwiching, “onion-skin” concentric lamination, tangential entrapment, and neural permeation [6]. Moreover, PNI is more easily recognized when occurring beyond the primary tumor (peripheral to the tumor), whereas its identification within the tumor mass is more challenging. To standardize diagnosis, some propose a 33% threshold for circumferential tumor involvement or direct tumor infiltration into any of the three layers of the nerve sheath, based on analyzing clinical samples from patients with pancreatic ductal carcinoma and head and neck squamous cell carcinoma, as well as experimental animal models [6,7,8]. This definition helps distinguish true invasion from focal abutment. Beyond the histological threshold, the underlying mechanism of PNI is thought to be the result of a complex interplay, or “crosstalk”, between tumor cells and the nervous system where neurotrophic factors and chemokines facilitate the migration of tumor cells along the nerve [3]. This bi-directional dynamic contributes to tumor spread and has been linked with poor prognosis in patients with certain cancer types [9].

That being said, and given its clinical impact and prognostic implications, it is important to distinguish between PNI and perineural entrapment. Perineural entrapment involves tumor cells abutting or compressing nerves without invasion, unlike PNI, which entails active tumor–nerve microenvironment interaction [10]. For instance, we observed and reported perineural entrapment in a 73-year-old woman with a left parotid gland mass, which was subsequently diagnosed as sclerosing polycystic adenoma—a benign neoplastic entity—upon histologic examination [11]. For pathologists, accurate differentiation of these processes is critical to ensure appropriate staging and optimal therapeutic management, as true PNI can represent a more aggressive tumor phenotype, while perineural entrapment may be a resulting effect or passive abutment of tumor expansion without evidence of tumor–nerve communication [1]. In breast cancer, PNI occurs in both benign and malignant lesions, though its clinical relevance remains under debate.

## 2. Historical Perspectives on Perineural Invasion in Breast Cancer

Breast cancer is the most prevalent non-cutaneous malignancy in the United States, with a lifetime risk of affecting approximately one in eight women by the age of 70 [12]. While surgical resection remains a cornerstone of curative treatment, the choice of adjuvant therapies, including chemotherapy and radiation, is primarily guided by the tumor’s clinicopathologic characteristics, which may involve presence or absence of PNI [13,14,15,16,17,18].

PNI was first noted in head and neck cancers by Cruveilhier and Neumann in the 19th century, observing tumor spread along nerves toward the intracranial fossa [19,20]. Since then, PNI has been recognized as a significant pathological feature in various malignancies, including those of the pancreas, colon, rectum, prostate, biliary tract, and stomach. In many of these cancers, PNI serves as an indicator of poor prognosis and is associated with reduced patient survival [21,22,23,24,25,26,27,28]. While PNI is well characterized in neurotropic cancers, its recognition in breast cancer has developed more gradually. In breast cancer, early studies on PNI primarily linked it to invasive tumors [1]. However, subsequent research challenged this notion. Ackerman documented the first case of nerve invasion in a benign breast lesion in 1957 [29]. A decade later, Taylor and Norris identified epithelial invasion of nerves in benign breast conditions, such as fibrocystic changes, sclerosing adenosis, and intraductal papillomatosis, in 20 out of 1000 consecutive breast biopsy cases [30]. Since then, multiple studies have reported similar findings, including PNI in radial scars and atypical ductal hyperplasia (ADH), among other benign breast conditions [2,10,31].

Research on PNI has primarily focused on cancers with a strong neurotropic tendency, such as pancreatic and prostate adenocarcinomas, whereas breast cancer has historically been characterized by its primary dissemination through lymphatic and vascular channels [1]. The complexity of characterizing and identifying PNI in breast cancer may have contributed to its underrepresentation in pathological reporting.

## 3. Prevalence and Clinicopathological Correlates of Perineural Invasion in Breast Pathology

Despite varying definitions of PNI, it has been extensively studied and recognized as an important prognostic marker in malignancies such as pancreatic [25,32], colorectal [1,23], prostate [33,34] and head and neck [35,36] cancers, where it is associated with decreased patient survival due to high recurrence rates and therapy resistance [3].

**Figure 1:**
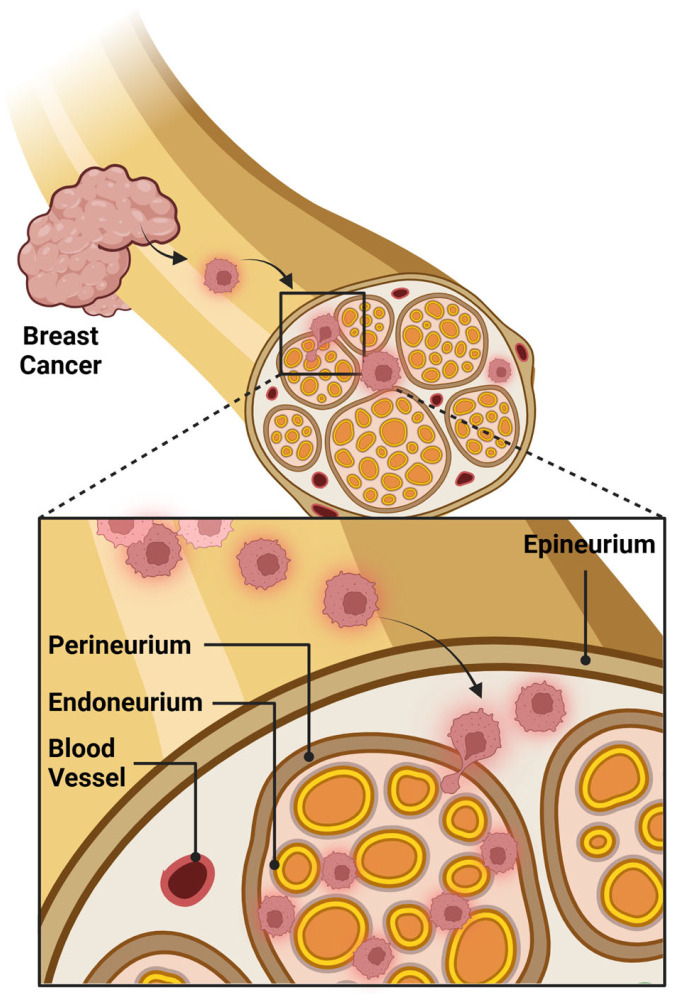
Schematic illustrating perineural invasion in breast cancer where tumor cells invade the perineum of peripheral nerves within the breast parenchyma. Created in BioRender. Bahmad, H. (2025) https://BioRender.com/i49c939.

In breast pathology, PNI has been identified and reported in both benign and malignant conditions [2,16,30,31,37] (Figure 1), yet its independent prognostic role is debatable. While a collective appreciation of its pathophysiology is emerging, lack of standardized diagnostic criteria and underreporting may have hindered efforts to define its frequency and clinical impact in breast pathology [1].

### 3.1. Perineural Invasion in Pre-Malignant and Benign Breast Lesions

The first reported case of benign breast PNI was documented by Ackerman in 1957, marking the initial recognition of this phenomenon outside of malignancies [29]. Further research has strengthened this finding, where PNI has been reported in ADH, ductal carcinoma in situ (DCIS), florid hyperplasia, sclerosing adenosis, and radial scars (Table 1). Gobbi et al. (2001) provided substantial evidence of PNI prevalence in non-invasive breast pathologies. Examination of 10,000 breast cases, excluding invasive mammary carcinoma, identified 14 cases of PNI within a distribution of specific patterns: five cases of ADH, three cases of DCIS (cribriform and comedo types), five cases of florid hyperplasia, and one case of ductal adenoma [31]. Nine cases developed within complex sclerosing lesions or radial scars (CSL/RS), four in sclerosing adenosis (SA), and one at a prior biopsy site of ductal adenoma [31]. These findings highlight the occurrence of PNI in non-invasive breast lesions, aligning with Taylor and Norris’s (1967) study, reporting PNI in 20 out of 1000 sclerosing adenosis cases (2%) [30]. Follow-up for 17 of those 20 patients was available, and all were well (median, 7 years) [30].

Additionally, Davies (1973) identified PNI in four out of 316 benign breast lesions (1.3%) [37]. Research has also identified PNI in cases involving cystic lesions, papillomatosis, oncocytic metaplasia, and periductal inflammation, where epithelial cells were observed involving nerve bundles [30,31,37]. Among these, radial scar, papillomatosis, intraductal papillomas, and sclerosing adenosis exhibited a higher propensity for PNI [30,37,38,39,40,41], further supporting the occurrence of nerve invasion beyond invasive carcinoma. A detailed histological evaluation by Elfituri and Emmadi (2019) also provided noteworthy insight into the complexity of PNI presentation in ductal carcinoma in situ (DCIS) PNI with multiple architectural patterns—cribriform, papillary, micropapillary—accompanied by findings of microcalcifications and focal necrosis [10,42]. Similarly, another case report that shows PNI presence in a benign breast condition is that of Fellegara et al. (2007), which described a case of florid adenosis [2].

**Table 1:** Cases with perineural invasion in benign breast diseases and ductal carcinoma in situ reported in the literature.

References	Total Cases Studied	Lesion Associated with PNI	Benign Breast Disease	DCIS	PNI Cases	PNI Prevalence (%)	Follow Up	Level of Evidence *
Ackerman, 1957 [29]	1	Benign breast disease	1	-	1	-	-	5
Taylor and Norris, 1967 [30]	1000 breast biopsies coded as sclerosing adenosis	Sclerosing adenosis	20	-	20	2.00%	Follow-up for 17 of 20 patients was available; all were well (median, 7 years)	2
Davies, 1973 [37]	316 mastopathies (excluding carcinomas)	Benign breast disease	4	-	4	1.30%	Patients were well after 8, 13, 28, and 38 months of follow-up	2
Gould et al., 1975 [38]	2	Extensive adenosis and papillomatosis	2	-	2	-	-	4
Tsang and Chan, 1992 [42]	1	DCIS (cribriform type)	-	1	1	-	-	4
Cerilli and Fechner, 2000 [40]	1	Intraductal papillomas with superimposed hyperplasia	1	-	1	-	Patient was well after 5 months of follow-up	4
Gobbi et al., 2001 [31]	10,000 breast biopsies (excluding carcinoma)	DCIS (cribriform and comedo types) and benign breast disease	11	3	14	0.14%	-	2
Doyle et al., 2007 [39]	125	Radial scars	4	-	4	3.20%	-	2
Fellegara and Kuhn, 2007 [2]	1	Florid adenosis	1	-	1	-	-	4
Chan and Chen, 2009 [41]	1	Fibrocystic changes with ductal hyperplasia and stromal sclerosis	1	-	1	-	Patient was well after 31 months of follow-up	4
Elfituri and Emmadi, 2019 [10]	1	DCIS (cribriform, papillary and micropapillary types)	-	1	1	-	Patient was well after 20 months of follow-up	4
Total	-	-	45	5	50	0.14–2.00%	-	-

Abbreviations: DCIS: ductal carcinoma in situ; PNI: perineural invasion. * Levels of evidence according to the updated Oxford Centre for Evidence-Based Medicine (OCEBM)—based on study designs—published in 2011: Level 1 includes high-quality systematic reviews or randomized controlled trials (RCTs); Level 2 comprises individual RCTs or observational studies with strong effects; Level 3 includes non-randomized cohort or follow-up studies; Level 4 refers to case series, case reports, and case-control studies; and Level 5 represents expert opinion or evidence based on theoretical reasoning without direct clinical data [43,44].

### 3.2. Perineural Invasion in Breast Cancer

There is considerable variability in the documented rate of PNI in breast cancer [45,46,47] (Table 2). Karak et al. (2010) reported low PNI rates of 1.14% [48], while Duraker et al. (2006) reported higher rates, as high as 25.73% [49]. This difference may be due to the variability in tumor type, cohort size, and patient population [50]. Specifically, in Karak’s study, PNI was identified in a relatively low percentage (1.14%; 13 of 1136 patients) of invasive mammary carcinoma cases, of which almost half (5/13) of the patients with PNI also had lymphovascular invasion (LVI). Results from this study demonstrated that PNI occurred 10 times less frequently than LVI, yet both PNI and LVI were associated with aggressive tumor features such as higher T-stage and intermediate to high histologic grade [48]. Nevertheless, due to the limited number of PNI-positive cases, establishing PNI as an independent prognostic factor was not feasible [48]. In Duraker’s study, a relatively high frequency of PNI (25.73%) was reported in invasive breast carcinomas (IBCs), with an even higher prevalence (up to 68.7%) observed in more aggressive pT4 tumors [49]. Despite this, the analysis found no significant association between PNI and disease-free survival, LRR, or distant metastasis. Furthermore, no correlation was observed between PNI and age, menopausal status, or estrogen receptor (ER) status [49].

### 3.3. Perineural Invasion Across Breast Cancer Subtypes

The incidence and distribution patterns of PNI across breast cancer subtypes demonstrate significant variability in current medical literature, with comprehensive subtype-specific analyses remaining limited [10,49,51] (Table 2). Certain breast cancer subtypes appear to have a higher propensity for PNI. Duraker et al. (2006) reported a higher frequency of PNI with ductal and mixed-type carcinomas, suggesting a correlation between PNI and aggressive histological patterns, with PNI observed in 25.7% of IBC cases [49]. Additionally, hormone receptor-positive tumors, particularly those expressing ER and/or progesterone receptor (PR), exhibited higher rates of PNI [49].

Hosoya et al. (2023) showed that invasive carcinoma of no special type (NST) showed a PNI incidence of 14.1%, while invasive lobular carcinoma (ILC) had a slightly lower rate at 13.3%, with no statistically significant difference between them. Notably higher rates were observed in mixed carcinomas and invasive micropapillary carcinomas, with incidences of 33.3% and 50%, respectively. Metaplastic carcinoma demonstrated the highest PNI incidence, observed in 100% of cases evaluated. In terms of molecular subtypes, HER2-positive tumors had a PNI incidence of 14.3%, whereas triple-negative breast cancers (TNBC) exhibited the lowest rate among subtypes, at 7.1%. These findings highlight the variability of PNI prevalence across different histologic and molecular breast cancer subtypes, suggesting that tumor biology may influence PNI [51].

**Table 2:** Prevalence, clinicopathological associations, and prognostic implications of perineural invasion in invasive breast carcinoma.

Reference	Cohort Size	PNI Cases	PNI Prevalence (%)	Clinicopathological Correlates of PNI	Prognostic Significance	Level of Evidence ^#^
Roses et al., 1982 [52]	122 patients with breast cancer (pT1; T1N0M0)	17/122	13.93%	Not assessed	PNI was not a significant predictor of LRR	2
Mate et al., 1986 [53]	180 patients with clinical stage I or II operable invasive mammary carcinoma treated by radiotherapy following local tumor excision	12/151	7.95%	Not assessed	PNI was not a significant predictor of LRR, although neither did LVI or tumor grade	2
McCready et al., 1996 [54] *	293 patients with primary invasive breast carcinoma s/p lumpectomy and negative margins	Not assessed	Not quantified	Not assessed	LVPI was a significant predictor of LRR	2
McCready et al., 2000 [55] *	244 patients with breast carcinoma s/p lumpectomy alone without adjuvant radiotherapy or adjuvant systemic therapy	LVPI in 82/229	LVPI prevalence 35.82%	Not assessed	LVPI was a significant predictor of LRR	2
Duraker et al., 2006 [49]	377 patients with invasive mammary carcinoma	97/377	25.73%	PNI positivity was 13.9% in pT1 tumors and 69.7% in pT4 tumors PNI higher in ductal and mixed type carcinomas than in other histologic types PNI associated with LVI, axillary lymph node involvement, and PR positivity	PNI was not prognostic for disease-free survival	2
Karak et al., 2010 [48]	1136 patients with invasive mammary carcinoma	13/1136	1.14%	PNI occurs 10 times less frequently than LVI PNI is associated with higher T-stage, tumor grade, and LVI	Patients with PNI can expect a meaningful survival at 5 years with appropriately aggressive adjuvant therapy (only 1 of the 13 patients died after mean follow-up of 5.9 years)	2
Koca et al., 2013 [56]	218 patients with pN3a breast cancer	20/218	9.17%	Not assessed	PNI was significantly associated with DFS (HR 2.519)	2
Narayan et al., 2021 [16]	8864 patients with invasive mammary carcinoma	1384/8864	15.61%	Not assessed	PNI independently predicted LRR after breast-conserving surgery Significant association between PNI and LRR (HR 1.46, *p* = 0.034)	2
Hosoya et al., 2023 [51]	191 patients with invasive mammary carcinoma s/p surgical resection	27/191	14.14%	PNI significantly correlated to larger tumor size, lymph node metastasis, and LVI Prevalence of PNI varies across histologic subtypes	PNI had a significant adverse effect on DMFS and DSS	2
Barb et al., 2023 [57]	229 cases (Romania, FFPE analysis)	Not assessed	Not quantified	PNI varied by TLS presence and subtype. TLS -ve Luminal B breast cancer: PNI more frequent in elderly and postmenopausal women; significantly associated with LVI TLS +ve Luminal B breast cancer: no PNI correlation Luminal B-HER2: PNI only in TLS -ve tumors TNBC-TLS +ve: PNI correlated with recurrence TNBC-TLS -ve: PNI strongly associated with high grade and recurrence.	Absence of TLS, stromal vascular features, and subtype-specific effects drive PNI and recurrence PNI most frequent in TLS-ve tumors Microvascular maturation inversely related to PNI in TNBC	2

Abbreviations: DFS: disease-free survival; DMFS: distant metastasis-free survival; DSS: disease-specific survival; LRR: locoregional recurrence; LVI: lymphovascular invasion; LVPI: lymphovascular or perineural invasion; PNI: perineural invasion; PR: progesterone receptor; s/p: status-post; TLS: tertiary lymphoid structures; TNBC: triple-negative breast cancer. * LVI and PNI were consolidated into a single risk factor (LVPI). ^#^ Levels of evidence according to the updated Oxford Centre for Evidence-Based Medicine (OCEBM)—based on study designs—published in 2011: Level 1 includes high-quality systematic reviews or randomized controlled trials (RCTs); Level 2 comprises individual RCTs or observational studies with strong effects; Level 3 includes non-randomized cohort or follow-up studies; Level 4 refers to case series, case reports, and case-control studies; and Level 5 represents expert opinion or evidence based on theoretical reasoning without direct clinical data [43,44].

## 4. Pathophysiology and Mechanisms of Perineural Invasion

### 4.1. Tumor Microenvironment

Extracellular matrix (ECM) stiffening is a well-documented factor that drives malignancy and progression of multiple tumors, including pancreatic ductal adenocarcinoma, hepatocellular carcinoma, and breast cancer [50,58]. Increased ECM stiffness has been shown to promote several malignant behaviors of cancer cells, such as enhanced proliferation, metabolic reprogramming, epithelial–mesenchymal transition (EMT), metastasis, and resistance to chemotherapy [59,60,61,62]. Recent studies demonstrate that a stiffened ECM significantly enhances the invasive capabilities of breast cancer cells [63]. Additionally, ECM stiffness alters the secretory profile of breast cancer cells, increasing nerve growth factor (NGF) expression, further facilitating interactions between cancer cells and nerve structures, ultimately promoting PNI [50].

Recent research by Han et al. (2024) explored the relationship between tumor microenvironment (TME) and PNI in breast cancer [50]. Authors demonstrated that a stiff ECM facilitates PNI via integrin β1-mediated signaling pathways. They found that PNI-positive tumors exhibit increased activation of integrin β1, focal adhesion kinase (FAK), and Yes-associated protein (YAP), features associated with aggressive breast cancer behaviors, thereby supporting the clinical observations of poorer outcomes in PNI-positive cases [50]. In other words, integrin β1 mediates mechanotransduction from ECM stiffness, activating FAK and YAP to promote PNI. Moreover, the type of nerve involved is of relevance, with sympathetic nerves primarily promoting tumor growth, and parasympathetic and sensory nerves exerting inhibitory effects on breast cancer progression [9].

Integrins, a family of heterodimeric cell-adhesion molecules, play a critical role in cellular interaction with the ECM [64,65]. They establish a direct mechanical link between the cell and its surrounding microenvironment by binding extracellular ligands and the intracellular cytoskeleton [64,65]. Among these, integrin β1 has been identified as the primary receptor responding to ECM stiffness in breast cancer. The increase in ECM rigidity enhances integrin-mediated mechanotransduction, intensifying tumor malignancy. Clinically, human breast tumors are characterized as exhibiting a fibrotic and stiffened microenvironment, and breast cancer cells within these tumors demonstrate increased integrin activity, focal adhesion formation, and integrin signal transduction. Furthermore, data derived from clinical samples support the experimental findings, indicating a strong correlation between breast tumor stiffness and the frequency of PNI [50].

Previous studies have established that tumor cells can activate downstream signaling cascades through ECM-binding receptors, including integrins, epithelial discoid protein domain receptors DDR1/2, mechanically sensitive channels PIEZO1/2, and the hyaluronic acid receptor CD44 [66,67,68]. These receptors play key roles in converting mechanical signals from the ECM into intracellular pathways, affecting tumor cell behavior and disease progression. Matrix crosslinking and increased stiffness enhance integrin signaling, fostering tumor progression and invasiveness [64]. Given these findings, targeting integrin-mediated mechanotransduction and associated signaling pathways presents a potential therapeutic opportunity to mitigate breast cancer progression and PNI [63,69,70,71,72].

Conclusively, recent evidence suggests that ECM stiffness not only promotes tumor cell invasion and innervation but may also modulate immune cell dynamics within the TME. A stiffened ECM has been shown to impair immune cell infiltration, particularly affecting T-cell trafficking and activation, potentially altering the immunological landscape required for tertiary lymphoid structure (TLS) development. The resulting immunosuppressive milieu may hinder TLS formation or function, thus diminishing local antitumor immunity and permitting enhanced perineural spread. Additionally, mechanosensitive pathways activated by ECM rigidity, including the YAP/TAZ and FAK axes, may indirectly suppress chemokine gradients and stromal interactions essential for TLS maturation.

### 4.2. Tertiary Lymphoid Structures

Tertiary lymphoid structures (TLSs) are ectopic lymphoid formations that arise in response to chronic inflammation and contribute to local antitumor immunity. Their presence and interaction with stromal blood vessels influence tumor behavior, including PNI and LVI [57,73,74,75]. TLS absence in HER2-positive and TNBC correlates with increased PNI, suggesting a protective role against perineural spread. HER2+/TLS- tumors exhibit a significant increase in PNI, and TNBC/TLS- tumors show the higher risk of recurrence and invasion [57]. These findings suggest that TLSs may play a protective role in limiting tumor progression and invasion.

The influence of TLSs on tumor invasion is closely related to the TME, as their interaction with stromal blood vessels varies among breast cancer subtypes, affecting immune cell recruitment, vascularization, and metastatic potential [57]. The interwoven connection between immune and stromal cells within TLSs is critical for their antitumor functions [76]. Despite their association with higher tumor grades and LVI, TLSs also correlate with improved disease-free survival in HER2+ breast cancers, suggesting their dual role in tumor aggressiveness and immune surveillance [77].

The prognostic significance of TLSs is further supported by their association with improved disease-free and overall breast cancer survival rates [78]. For instance, one study found that TLSs are associated particularly with early-stage tumors and high tumor-infiltrating lymphocytes. TLSs’ presence suggests a strong antitumor immune environment, reinforcing their potential as prognostic indicators in breast cancer [78].

The interplay between ECM stiffness and TLS formation suggests a complex crosstalk between stromal mechanics and immune regulation in breast cancer. It is plausible that in tumors with highly fibrotic and rigid ECMs—such as in triple-negative and HER2-positive subtypes, as previously discussed—the physical properties of the stroma may limit immune cell recruitment or positioning, thereby impairing TLS assembly. This could explain the observed correlation between TLS absence and increased PNI in these subtypes. Moreover, the absence of TLSs may further permit unchecked neurotrophic signaling and tumor–nerve interactions, creating a permissive environment for neural infiltration. Thus, the convergence of biomechanical and immunological dysregulation may jointly facilitate perineural invasion in aggressive breast tumors.

### 4.3. Molecular Pathways

Cancer innervation refers to the recruitment of nerves by tumors into the TME and the induction of nerve branches within the TME, which in turn affects cancer development [79,80]. Tumor innervation is considered a critical factor in cancer progression, as nerves regulate various biological processes within the TME, including cellular proliferation, immune evasion, and metastatic potential.

Regarding the origin of these nerves, several studies have reported that tumors can recruit or modify peripheral nerves to support their growth [79,81]. This suggests an intricate interaction between cancer cells and the nervous system, enabling tumors to utilize neural signaling for their advantage. Specifically, tumor cells can induce axonal sprouting and neurogenesis, further incorporating nerve fibers into the TME [82].

Pathological specimens reveal a higher density of nerve fibers in breast cancer tissue compared to normal breast tissue or benign breast tumor tissue, with a positive correlation between nerve density and tumor aggressiveness [83,84]. This association emphasizes the potential role that nerve infiltration may have in facilitating tumor progression [85]. The presence of these nerve fibers may serve as a prognostic indicator of patient outcomes.

Breast cancer cells can promote the intratumoral infiltration of nerve fibers, which further intensifies with the malignant progression of the disease. Several studies show that breast cancer cells secrete various cytokines, including neurotrophins and vascular endothelial growth factor (VEGF), to promote the growth and branching of nerve fibers [85,86,87]. Therefore, tumor-induced nerve growth is an active process driven by tumor-derived factors. The interaction between breast cancer cells and nerve fibers highlights a critical path in breast cancer pathology that may provide novel targets for therapeutic intervention.

## 5. Histological Features of Perineural and Intraneural Invasion in Breast Pathology

**Figure 2:**
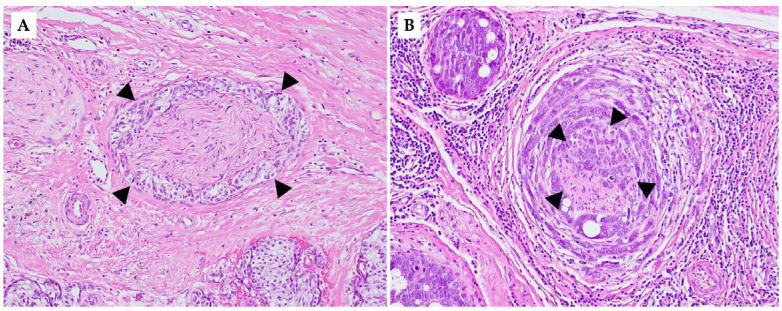
Representative hematoxylin and eosin (H&E)-stained image illustrating perineural and intraneural invasion in invasive ductal carcinoma. (**A**) Perineural invasion with tumor cells surrounding the nerve (arrowheads). (**B**) Intraneural invasion with tumor cells within the nerve fascicle (arrowheads) (200×).

Perineural and intraneural involvement in DCIS and IBC demonstrate significant histopathological findings (Figure 2). In DCIS, tumor cells are present in the surrounding nerve sheath without invasion into the nerve itself. It has been shown that PNI in the context of DCIS often occurs in a background of complex sclerosing/radial scars and sclerosing adenosis, as mentioned previously, which can be a key histopathologic indicator of DCIS [31]. By definition, DCIS is non-invasive; intraneural invasion is exclusive to invasive carcinomas. Therefore, compared to PNI, intraneural invasion involving DCIS is not possible, emphasizing intraneural invasion as a characteristic of IBC.

Histological analysis often demonstrates displacement of epithelial glands into the perineural space closely mimicking true invasion and further complicating accurate pathological assessment through misinterpretation as invasive carcinoma. This emphasizes the need for increased awareness among pathologists regarding the potential for PNI in ADH and DCIS. Careful histopathological evaluation, along with correlation with clinical and radiologic findings, is essential to avoid misdiagnosis. In rare challenging cases, the use of immunohistochemical (IHC) analysis can be instrumental to help differentiate between DCIS and IBC and ensure accurate pathological diagnosis [42]. In DCIS, the myoepithelial layer remains intact, whereas in invasive carcinoma, it is disrupted or lost [10]. So IHC markers like p63 or calponin may be used to confirm myoepithelial integrity in DCIS, distinguishing it from invasive carcinoma. While IBC can demonstrate features of PNI similar to DCIS, the key defining feature in intraneural invasion includes the presence of tumor cells within the nerve fascicle [42]. Specifically, infiltration into the endoneurium and axonal degeneration secondary to tumor cell invasion are key features.

Histologically, PNI is identified based on standard hematoxylin and eosin (H&E)-stained sections, where tumor cells are seen tracking along, wrapping around, or infiltrating nerves. There are no IHC stains currently validated or routinely used to predict PNI in any type of cancer, including breast cancer. While certain IHC markers (e.g., S100 and neurofilament proteins) may help highlight nerve fibers in challenging cases (for morphologic confirmation when nerves are hard to distinguish) [88,89], they are not used to predict PNI. In terms of predictive biomarkers, as discussed previously, studies have identified molecular markers (e.g., neurotrophins, MMPs, RET, etc.) associated with PNI, but none are currently used clinically in routine pathology.

## 6. Reporting Perineural Invasion in Breast Cancer

Given the emerging research on PNI as a potential indicator of aggressive tumor behavior, standardizing PNI reporting especially in breast cancer could improve risk stratification. The College of American Pathologists (CAP) includes the assessment of PNI within its standardized guidelines for evaluating certain malignancies, such as cutaneous squamous cell carcinoma, prostate, gastric, colorectal, gallbladder and pancreatic ductal adenocarcinoma [23,24,25,26,34,36,90]. However, unlike other malignancies, the CAP protocol for breast cancer does not mandate PNI reporting, reflecting its uncertain prognostic value [91]. This may reflect the inconsistencies in its detection and interpretation but equally highlights the need for additional research to bridge the gaps. Nonetheless, accurate documentation and incorporation of PNI in pathology reports will undoubtedly contribute to awareness of it and support clinicians in optimizing risk assessment.

## 7. Imaging Modalities for Perineural Invasion Detection in Breast Cancer

Imaging modalities play a vital role in PNI diagnosis, including magnetic resonance imaging (MRI), positron emission tomography (PET), and computed tomography (CT). For detecting PNI in breast cancer, especially with suspected brachial plexus involvement, MRI remains the gold standard due to its superior soft tissue contrast and ability to visualize neural structures. These characteristics enhance MRI sensitivity for detecting PNI in breast cancer subtypes where perineural spread is suspected. Doran et al. (2024) described key MRI features indicative of PNI, including T2 signal expansion, abnormal enhancement, and loss of normal fat planes surrounding nerve pathways [92]. In head and neck tumors, MRI can detect perineural tumor spread with a sensitivity of 95%, though its accuracy decreases to 63% when mapping the full extent of the spread [93]. PET using 18F-fluorodeoxyglucose (FDG) serves as an adjunct imaging technique to MRI, detecting abnormal FDG accumulation along nerve pathways in a linear distribution. This pattern is highly suggestive of PNI, and when combined with MRI, enhances the accuracy of PNI detection [92]. A CT scan is a secondary modality utilized for detecting PNI due to its decreased sensitivity compared to MRI. Tu et al. (2023) reported common CT findings, including abnormal soft-tissue attenuation extending along neural structures. However, CT’s low contrast resolution limits its effectiveness in accurately delineating nerve involvement [94].

While imaging provides vital diagnostic and pathologic insight, limitations exist. PNI can mimic other conditions, including radiation-induced neuritis or chemotherapy-associated neuropathy [95]. PNI may present with minimal abnormalities on MRI, such as intermediate T2 signal expansion or subtle abnormal enhancement extending along nerve pathways. These features create difficulty in detection without high-resolution imaging and careful interpretation [96].

Post-therapy changes further complicate PNI detection, as prior surgical interventions or radiation therapy may distort anatomic structures, obscuring the presence of nerve invasion. This is particularly relevant in treatment-related fibrosis and scarring, common with breast cancer, which can mimic or mask perineural tumor spread [95]. Technical limitations also pose additional challenges. High-resolution thin-cut contrast-enhanced MRI is optimal for detecting PNI, but specialized imaging may not be available in all clinical settings. Emerging techniques, including tractography and contrast-enhanced PET/MRI, hold promise for improving PNI detection but remain under investigation and are not yet widely accessible [97].

## 8. Clinical Manifestations of Perineural Spread in Breast Cancer

In breast diseases, the clinical utility of imaging modalities is exemplified in case reports demonstrating their role in diagnosing perineural tumor spread.

### 8.1. Brachial Plexopathy from Perineural Spread

The perineural spread of breast cancer to the brachial plexus is a rare but increasingly recognized phenomenon. Anatomical studies and clinical reports provide insight into pathways and manifestations. The pathways through which breast cancer metastasizes to the brachial plexus remain uncertain. However, proposed mechanisms describe PNI via longitudinal extension of neoplastic cells within the brachial plexus, occurring in both anterograde and retrograde directions. This allows for tumor spread to the nerves of the arm, cervical roots, spinal canal, spinal fluid, and contralateral brachial plexus [96]. Anatomical studies suggest the intercostobrachial nerve is the most likely route, given its proximity to the breast and established communication with the brachial plexus. Established communications include the medial cord, medial and posterior antebrachial cutaneous nerves, and the T2 ventral ramus [96].

Other potential, less common routes include the lateral and medial pectoral nerves, arising from the lateral and medial cords, and supraclavicular nerves, which may communicate with the brachial plexus via the suprascapular nerve and C5 root [96]. Murthy et al. (2020) further support this hypothesis, suggesting that malignant cells invade small nerves with connections to the brachial plexus, such as the intercostobrachial, pectoral, and supraclavicular nerves, before spreading intraneurally to parent nerves and entering the brachial plexus [98]. Given that most breast cancers originate in the upper outer quadrant, the intercostobrachial nerve is considered the most probable pathway for perineural spread [98].

Loukas et al. (2006) described extrathoracic communication with the brachial plexus involving T2 and the intercostobrachial nerve. It was suggested that extrathoracic communications arise via the intercostobrachial nerve and medial cord and intrathoracically through the ventral ramus of T2, serving as conduits for perineural tumor spread [99]. This theory was demonstrated in a case report involving a 51-year-old woman with a history of ER-positive IDC of the breast who developed progressive left brachial plexopathy more than 20 years following treatment [96]. Her symptoms evolved from numbness and pain in the medial arm to weakness, Horner’s syndrome, and contralateral involvement, suggestive of bilateral PNI [96]. MRI revealed diffuse thickening and nodular enhancement of the left brachial plexus, extending into the intradural C7 and C8 roots. PET/CT showed increased metabolic activity in the left brachial plexus. A fascicular biopsy of the left lower trunk confirmed metastatic adenocarcinoma within the nerve, with strong ER positivity and HER2/neu amplification [96].

Retrospective studies have further documented perineural spread of breast cancer to the brachial plexus. A study by Jack et al. (2022) reviewed 19 patients with biopsy-confirmed perineural spread [100]. Im et al. (2024) described seven patients with brachial plexopathy secondary to perineural tumor spread, identifying IDC (3), metaplastic carcinoma (1), lung adenocarcinoma (2), and papillary thyroid carcinoma (1) as primary malignancies. Initial presentation involved progressive unilateral pain or paresthesia, followed by motor weakness, with lower trunk plexopathy being the most common electrodiagnostic finding [101].

### 8.2. Intradural Extramedullary Metastasis via Perineural Invasion

Rare cases of intradural extramedullary metastasis via PNI illustrate the potential for breast carcinoma to metastasize to the spinal cord and nerve root ganglia. Schulz et al. (2009) reported a case of breast cancer metastasis to the L2 nerve root ganglion, involving intradural tumor spread. A 46-year-old female presented with severe right-sided radicular pain and moderate weakness of hip flexion and knee extension. MRI revealed an L2 nerve root lesion, followed by histologic examination confirming breast cancer metastasis via immunostaining [102].

Jayakumar et al. (2020) described a woman in her late sixties with breast cancer and pre-existing infiltrative disease of the left brachial plexus. Spinal MRI revealed an intramedullary spinal cord metastasis at C6-C7. This case highlights intramedullary metastases with the potential for PNI extending beyond brachial plexus lesions into the spinal cord [103].

Similarly, Mackel et al. (2020) reported a 66-year-old woman with a history of node-positive ER/PR-positive infiltrating ductal carcinoma, who developed bilateral buttock and leg pain, twelve years later. MRI of the total spine revealed a bilobed, intradural, intramedullary–extramedullary, homogenously enhancing lesion involving the conus medullaris and cauda equina. The lesions confirmed suspicion of intradural metastasis via PNI, secondary to prior breast carcinoma. This case further emphasizes the delayed and unpredictable nature of perineural metastatic spread in breast cancer, as well as the devastating consequences associated with extension to the spinal cord and nerve roots [104].

### 8.3. Optic Nerve Sheath Metastasis

Optic nerve sheath metastasis, though rare, presents with vision loss and perineural enhancement on MRI. A large study by Yousef et al. (2024) analyzed 9902 breast cancer patients, reporting that 0.5% developed ocular or periocular metastasis, which accounted for 2.4% of all metastatic cases. Further breakdown displayed optic nerve involvement in 11% of those cases, with 44% of patients exhibiting bilateral disease. The time of ocular metastasis varied, with 49% occurring after breast cancer diagnosis, 24% occurring concurrently, and 5% appearing before primary tumor diagnosis. The most common sites of ocular metastasis included the orbit (47%), choroid (40%), and iris (2%) [105].

Gasperini et al. (2007) reported a 36-year-old woman with bilateral optic neuropathy and central nervous system metastasis from breast carcinoma. Brain and orbital MRI revealed diffuse meningeal enhancement, perineural enhancement of both optic nerves, and enhancement of the right optic nerve head, consistent with metastatic disease [106].

Similarly, Verma et al. (2019) described a 68-year-old woman with a history of breast cancer and bone metastases who presented with blurring in her left eye two decades after mastectomy. Examination revealed an isolated peripapillary mass, with MRI confirming optic disc metastasis secondary to breast carcinoma [107].

In another case, Biswas et al. (2007) reported a 39-year-old woman with a known history of breast cancer who developed bilateral painless vision loss. Examination revealed subretinal lesions, retinal detachment, and later left proptosis with ocular movement restriction, indicating extraocular metastatic spread. Histopathology confirmed lobular adenocarcinoma, highlighting the potential for optic nerve and retinal involvement in metastatic breast cancer [108].

## 9. Prognostic Significance of PNI in Breast Cancer

Given the wide range of PNI frequencies reported in breast cancer, its prognostic relevance remains unclear. Nevertheless, Liebig et al. (2009) highlighted an important consideration in that PNI is frequently reported jointly with LVI as one pathologic feature in breast cancer, clouding its value as an independent prognostic marker [1]. For instance, in two separate retrospective studies, McCready et al. (1996 and 2000) evaluated PNI in conjunction with LVI in IBC [54,55]. Findings from the two studies suggested an association between these combined features and increased risks of local recurrence and distant metastasis [54,55] (Table 2).

### 9.1. Perineural Invasion and Locoregional Recurrence

PNI has been studied in many different types of cancers, but there is still debate over its significance in breast cancer and whether it directly increases the risk of LRR or if it is just a marker of more aggressive tumors [16,52,53,54,55].

Narayan et al. (2021) found a higher 7-year LRR rate in PNI-positive cases (7.1% vs. 4.7%) in a cohort of 8864 IBC patients. After controlling for key clinical and pathological variables, the risk of LRR remained notably higher among patients with PNI (hazard ratio 1.46, *p* = 0.034), conferring an increased risk of recurrence, comparable in magnitude to LVI and ER/PR-negative receptor status. These findings reflect the possible prognostic impact of PNI in breast cancer [16].

### 9.2. Perineural Invasion Versus Lymphovascular Invasion

Several studies have examined the relationship between PNI and LVI in breast cancer, with differing conclusions on whether PNI independently affects prognosis. Narayan et al. (2021) [16] found that tumors with PNI were more likely to also have LVI, but PNI predicted worse outcomes, whereas LVI showed an association but had weaker hazard ratio and lower statistical confidence. Hosoya et al. (2023) confirmed a strong association between PNI and LVI and found that PNI independently predicted recurrence even when adjusting for LVI [51]. On the other hand, Duraker et al. (2006) observed a strong correlation between PNI and LVI but concluded that PNI was not an independent prognostic factor, and that LVI contributed more toward prognosis and survival whereas PNI was not statistically significant [49].

Therefore, although all three studies show a relationship between PNI and LVI, they ultimately have differing conclusions in terms of the role that PNI has on prognosis. While Narayan et al. (2021) and Hosoya et al. (2023) both support PNI as an independent prognostic factor even after LVI is adjusted for, Duraker et al. (2006) found it non-significant after adjusting for LVI [16,49,51]. These conflicting results may stem from several factors. First, the definition and histologic criteria used to diagnose PNI may vary across studies, potentially affecting consistency in reporting. Second, differences in sample size and patient cohorts—particularly in terms of tumor subtype distribution, stage at diagnosis, and treatment modalities—could influence statistical power and outcomes. Additionally, variations in how LVI is assessed and controlled for in multivariate models may impact the apparent independent contribution of PNI. That being said, there is a need for standardized diagnostic criteria and large-scale prospective studies to determine the true prognostic value of PNI in breast cancer.

## 10. Therapeutic Implications and Future Directions

### 10.1. Current Treatment Approaches

Breast cancer that presents with PNI has become an increasingly important feature when it comes to assessing tumor biology and patient prognostic factors with the aim of establishing effective therapeutic measures. The interaction of malignancies with the peripheral nervous system has been shown to be a crucial component in the progression of such neoplasms [9]. Furthermore, breast lesions such as ADH and DCIS of the breast, which may exhibit PNI, indicate the possibility of early involvement of the nervous system and its structures in tumor development [10,16,31].

Currently, the mainstay of treatment of breast cancer remains hormonal therapy, neoadjuvant chemotherapy, and surgical intervention [16,51]. Radiation may reduce LRR risk in PNI-positive cases, similar to its use in head and neck cancers [49]. While there is a strong association between PNI and cancer outcomes in other malignancies such as head and neck squamous cell carcinoma, prompting the use of radiation therapy to improve local control [109,110], a lack of enough resources has yet to indicate such an association in breast cancer [16]. Radiation therapy is indicated in breast cancer treatment after surgery and sometimes before surgery (neoadjuvant therapy) to reduce the risk of recurrence, particularly in cases with high-risk pathological features, such as lymph node involvement, large tumors (larger than 5 cm) which carry a higher risk of recurrence, positive or close surgical margins, and cancers with extensive LVI [111,112].

Current breast cancer therapies do not adjust for PNI status. Patients with TNBC may benefit from intensified chemotherapy regimens, such as anthracycline and taxane-based therapies, due to the aggressive nature of TNBC [113]. For HER2-positive subtypes, the addition of targeted therapies like trastuzumab is beneficial, despite the PNI status [114]. Hormone receptor-positive subtypes may require a combination of endocrine therapy and chemotherapy to address the potential for LRR.

### 10.2. Emerging Targets for Therapy

Current breast cancer therapies do not adjust for PNI status, but emerging data suggest targeted therapies could benefit PNI-positive cases. In fact, growing research regarding therapy for malignancies that are positive for PNI has led to several novel targets that may impact the process which drives PNI.

#### 10.2.1. Integrin Signaling and ECM Stiffness

Within this category of potential therapeutic methods, the role of the ECM and the role of integrins in the spread and invasion of PNI-positive tumors has been assessed [50]. Inhibiting integrin β1 or NGF signaling could disrupt PNI mechanisms, offering new therapeutic possibilities. It has been demonstrated by Han et al. (2024) that integrin signaling through B1-FAK-YAP is further promoted by a stiffened microenvironment within tumors [50]. The evidence highlighted indicates that possibly targeting integrin B1 could disrupt this cascade pathway and, in turn, reduce tumor invasion into neural structures [50]. Additionally, integrin B4 is another component that has shown expression not only in vascular and lymphatic structures, but also in peripheral nerves, which supports current research in using it as a staining marker compared to the more conventional H&E [68]. Research into this molecule can potentially reveal further possibilities in the development of therapeutic measures, in addition to detection methods currently being studied.

#### 10.2.2. Neurotrophic Factor Blockade

Adding on to other studied methods involving integrin signaling, the effective blockade of neurotrophic signaling has become a promising strategy in the treatment of PNI-positive breast cancer [115]. Nerve growth factor (NGF) has been reported to be a target for such therapy due to the associated overexpression of NGF in breast cancer cells when compared to other tissues such as breast epithelium [115]. This further indicates that an increase in NGF may be associated with further growth in breast cancer cells, and it has been discovered that effectively diminishing or neutralizing NGF contributed to reduced ability of malignant cell proliferation [115]. This indicates that if NGF reduction reduced malignant cell proliferation, then inhibiting NGF may provide improved outcomes in inhibiting tumor growth [115]. Additionally, further studies regarding neurotrophic factor blockades have indicated that anti-NGF antibodies can significantly reduce the neurotrophic effect of NGF and thus inhibit nerve fiber growth, which is a crucial component in PNI-positive breast cancers [85].

### 10.3. Need for Further Research

Understanding the molecular pathways for targeted interventions is also essential, as PNI is not considered a passive process anymore, but active biological crosstalk between nerves and malignant cells [116]. A key pathway being assessed within the neurotrophin receptor system is NGF-TrkA signaling which indicates that this pathway is a chief contributor for PNI [117]. This pathway is being increasingly researched due to higher TrkA expression being observed in IBCs [118], opening the possibility for this pathway being studied for the development of effective therapy [117]. Additionally, another pathway that would be crucial to further understanding PNI-positive breast cancer is the MAPK/ERK pathway. This is due to studies indicating that the MAPK/ERK pathways are involved in the signaling that contributes to malignant spread of cancer cells [119,120], possibly by contributing to their ability to affect the ECM around nerves.

## 11. Conclusions

Perineural invasion in breast pathology holds important clinicopathological implications. Its association with aggressive tumor behavior, potential locoregional recurrence, and possible poor prognosis highlights the need for improved recognition and reporting. Advances in molecular profiling have begun to elucidate the complex tumor–neural interactions that facilitate PNI, suggesting potential targets for therapeutic intervention. However, current knowledge remains limited due to the lack of standardized diagnostic criteria. Addressing these gaps through future research could refine risk stratification models and guide personalized treatment strategies. Recognizing the significance of PNI in breast cancer may ultimately contribute to better clinical management and patient outcomes. Therefore, future research should standardize PNI definitions, explore its molecular basis, and evaluate targeted therapies to improve outcomes in PNI-positive breast cancer.

## Data Availability

No new data were created or analyzed in this study. Data sharing is not applicable to this article.
